# Parental and carer views on the use of AI in imaging for children: a national survey

**DOI:** 10.1186/s13244-025-02021-6

**Published:** 2025-08-09

**Authors:** Girija Agarwal, Raimat Korede Salami, Lauren Lee, Helena Martin, Lahvanya Shantharam, Kate Thomas, Emily Ashworth, Emma Allan, Ka-Wai Yung, Cato Pauling, Deidre Leyden, Owen J. Arthurs, Susan Cheng Shelmerdine

**Affiliations:** 1 Imperial College Healthcare NHS Trust, London, UK; 2 Kings College Hospital NHS Foundation Trust, London, UK; 3 Young Persons Advisory Group (YPAG), Great Ormond Street Hospital for Children, London, UK; 4 Guy’s and St Thomas’ NHS Foundation Trust, London, UK; 5 St George’s Hospital, London, UK; 6 Victoria Hospital, NHS Fife, Kirkcaldy, Scotland, UK; 7 Department of Clinical Radiology, Great Ormond Street Hospital for Children, London, UK; 8 Wellcome/EPSRC Centre for Interventional and Surgical Sciences, Charles Bell House, London, UK; 9 University College London, London, UK; 10 UCL Great Ormond Street Institute of Child Health, Great Ormond Street Hospital for Children, London, UK; 11 NIHR Great Ormond Street Hospital Biomedical Research Centre, London, UK

**Keywords:** Surveys and questionnaires, Artificial intelligence, Parents, Medical imaging, Paediatric

## Abstract

**Objectives:**

Although the use of artificial intelligence (AI) in healthcare is increasing, stakeholder engagement remains poor, particularly relating to understanding parent/carer acceptance of AI tools in paediatric imaging. We explore these perceptions and compare them to the opinions of children and young people (CYAP).

**Materials and methods:**

A UK national online survey was conducted, inviting parents, carers and guardians of children to participate. The survey was “live” from June 2022 to 2023. The survey included questions asking about respondents' views of AI in general, as well as in specific circumstances (e.g. fractures) with respect to children’s healthcare.

**Results:**

One hundred forty-six parents/carers (mean age = 45; range = 21–80) from all four nations of the UK responded. Most respondents (93/146, 64%) believed that AI would be more accurate at interpreting paediatric musculoskeletal radiographs than healthcare professionals, but had a strong preference for human supervision (66%). Whilst male respondents were more likely to believe that AI would be more accurate (55/72, 76%), they were twice as likely as female parents/carers to believe that AI use could result in their child’s data falling into the wrong hands. Most respondents would like to be asked permission before AI is used for the interpretation of their child’s scans (104/146, 71%). Notably, 79% of parents/carers prioritised accuracy over speed compared to 66% of CYAP.

**Conclusion:**

Parents/carers feel positively about AI for paediatric imaging but strongly discourage autonomous use. Acknowledging the diverse opinions of the patient population is vital in aiding the successful integration of AI for paediatric imaging.

**Critical relevance statement:**

Parents/carers demonstrate a preference for AI use with human supervision that prioritises accuracy, transparency and institutional accountability. AI is welcomed as a supportive tool, but not as a substitute for human expertise.

**Key Points:**

- Parents/carers are accepting of AI use, with human supervision.
- Over half believe AI would replace doctors/nurses looking at bone X-rays within 5 years.
- Parents/carers are more likely than CYAP to trust AI’s accuracy.
- Parents/carers are also more sceptical about AI data misuse.

**Graphical Abstract:**

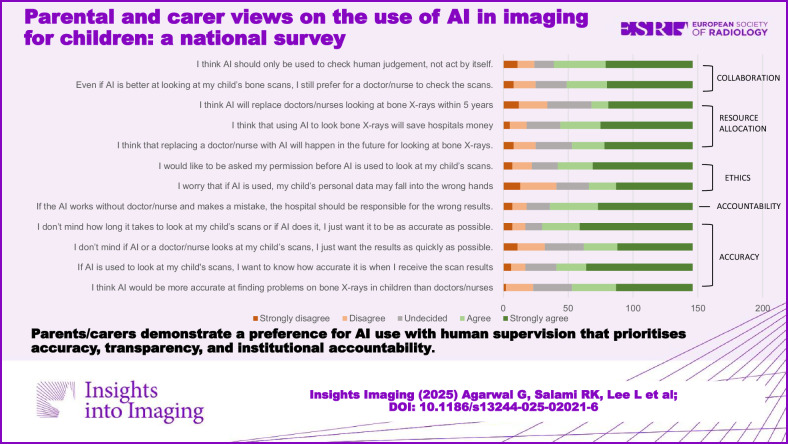

## Introduction

Stakeholder engagement is critical for the successful deployment and adoption of AI applications in healthcare [1]. It is especially important to understand what drives patient trust and acceptability, as these tools primarily impact their care. In paediatric radiology, stakeholder engagement not only needs to take the opinion of the patient, but must also consider the views of their parents or caregivers. Nevertheless, despite the importance of this factor, most published work has focused mostly on the perspectives of medical healthcare professionals [2, 3], with fewer studies assessing the attitudes of parents or caregivers [4, 5].

One survey which evaluated the views of caregivers of children who presented to an outpatient orthopaedic fracture clinic, on the use of AI for fracture detection on imaging, found the majority of caregivers (82%, 135/163) were happy with the idea of an AI being used to assist doctors or nurses, whereas 16/163 (10%) were not comfortable with any use of AI at all, either acting alone or assisting a medical professional [6]. A national survey of British children and young adults, evaluating children’s opinions on the use of AI for their imaging (with a focus on fracture detection) revealed that young patients prioritise the accuracy of AI tools over speed, emphasising the need for consent and transparency in AI usage, and a strong preference for AI assisting a ‘human in the loop’ rather than ‘AI acting alone’ [7].

In this study, we build upon this prior work by presenting the results of the same national survey, but detailing the responses and views of the parents and caregivers who took part. Understanding their perspectives, and importantly, how these differ from those of children and young adults, is crucial in ensuring that the implementation of AI for children’s fracture detection addresses the concerns of both stakeholders.

## Methods and materials

Ethical approval was not required for this voluntary questionnaire of public opinions.

### Questionnaire

The survey was based on a validated questionnaire developed by Ongena et al [8] regarding patient views on the implementation of AI in radiology. Their survey was developed by methodologists in collaboration with radiologists and 155 patients undergoing diagnostic imaging tests and consisted of six domains (i.e. proof of technology, procedural knowledge, competence, efficiency, personal interaction and accountability).

Our survey was built upon this validated survey as a basis for further discussion across three patient and public engagement meetings held between May and October 2021 by the ‘FRACTURE Study Patient and Public Involvement & Engagement (PPIE) Steering Committee’ [9]. This committee consisted of three parent representatives, four young person representatives (aged between 15-years old and 23-years-old), the institution’s PPIE Manager for research (DL), and the lead researcher for this study (SS). The parents and young people on this steering group were self-selected volunteers from two larger PPIE groups (called the ‘Great Ormond Street Hospital for Children London Young Persons’ Advisory Group’ for research (GOSH YPAG) and ‘Great Ormond Street Hospital for Children Parent and Carer Advisory Group for research) with an interest in digital technology [10]. Two versions of the survey (a child-friendly and adult-friendly version) were developed. The results from the child-friendly survey have been previously published [7]. The final adult version of the survey was tested amongst adult members of the steering committee to ensure user understanding.

To attract public attention and participation, an accompanying short animation [9] explaining what the survey was asking for and how to take part, was also developed in consultation with the steering committee. The final survey was conducted in English and hosted on the Google Forms platform.

The survey contained a total of 33 questions (ESM Supplementary Table S1) which comprised of 6 basic questions on demographic details (age, gender, ethnicity, location, caring situation with children, education), 2 questions about computer and AI experience/knowledge, then 2 questions about the respondents’ child’s fracture history.

We used a 5-point Likert scale to score 21 subsequent questions regarding preferences for use of AI in children’s imaging (12 questions were specific to the use of AI for musculoskeletal imaging and 9 relating to AI for cardiac, oncological and neurological imaging collectively), with another 2 open-ended responses for elaboration on opinions. Questions were categorised according to five themes (i.e. accuracy, responsibility, ethical issues, resource allocation, and collaboration).

### Dissemination

The survey was ‘live’ for a total period of 12 months from 1 June 2022 to 31st May 2023, and was disseminated through various local and national contacts, including:

- Emailing 200 UK primary and secondary school administrators (ensuring dissemination across the four UK nations and boroughs).
- Emailing YPAG (Young Person’s Advisory Group for research) Generation R representatives to share with their carers [10].
- Hosting the link on the FRACTURE Study website [9] and Twitter feed.
- Hosting the link on the Great Ormond Street Hospital website [11] and Twitter feed.
- Hosting the link on the Brittle Bone Society website [12] and Twitter feed.
- Hosting the link to the animation and survey on Mumsnet, a popular UK-based online forum designed for parents.
- Word of mouth and ‘retweeting’ of the survey link via local contacts within the GOSH YPAG group.
- Two email reminders were sent to local and national contacts during the study period.

### Data analysis

Simple descriptive statistics were used and analysed in Excel for Microsoft Office.

To determine if there were statistically significant differences between the responses of adults and those of children and young adults previously published by our research team [7], a Chi-square test of independence was employed. A *p*-value of less than 0.05 was considered statistically significant. Similar statistical methods were used for subgroup analysis performed with statistical software R.

## Results

### Respondent demographics (Table 1)

**Fig. 1:**
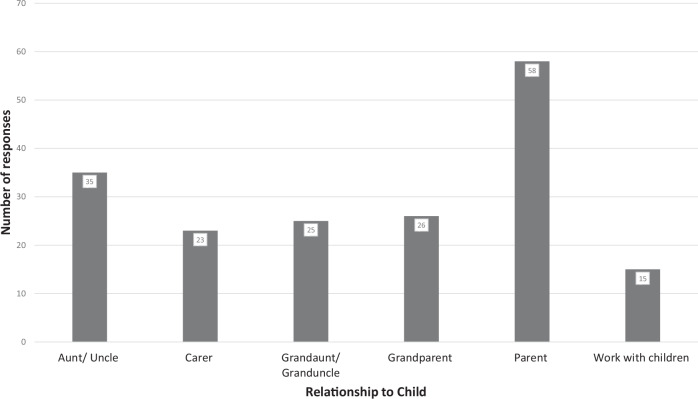
Bar chart demonstrating the relationships with children described by respondents, respondents were able to select more than one option in this survey (*n* = 146)

During the study period, 149 participants completed the survey. Three were excluded as they were 20 years old or younger and did not report a parent/carer relationship with a child. Participants who were included reported multiple co-existing roles in the lives of children, but most commonly parent (58/146, 40%) (Fig. 1). The majority of participants were in the 41–50 age group (49/146, 34%) (Fig. S1). There was an equal distribution of female and male respondents (both 72/146, 49%), and 2/146 (2%) preferred not to say.

**Table 1:** Demographic summary of respondents

Category	Subgroup	Count	Percentage of total
Gender	Male	72	49%
	Female	72	49%
	Prefer not to say	1	2%
Age (years)	21–30	19	13%
	31–40	34	23%
	41–50	49	34%
	51–60	31	21%
	61–70	12	8%
	71 and above	1	1%
Ethnicity	Asian/Asian British	38	26%
	Black/African/Caribbean/Black British	23	16%
	White/Caucasian	42	29%
	Mixed/Multiple ethnic groups	31	21%
	Prefer not to say	12	8%
Caring situation (multiple options could be selected, so the total is over 146)	Carer	23	16%
	Grandparent	26	18%
	Parent	58	40%
	Grandaunt/granduncle	25	17%
	Aunt/uncle	35	24%
	Work with children	15	10%
Highest level of education	Never attended formal education	6	4%
	Primary/secondary/high school	9	6%
	Associate	20	14%
	Undergraduate degree	39	27%
	Postgraduate degree	52	36%
	Professional degree (e.g. Medicine)	19	13%
	PhD	1	1%
Self-reported computer skills (score 1–5, where 1 is the lowest)	1	7	5%
	2	27	18%
	3	28	19%
	4	49	34%
	5	35	24%
Self-reported AI knowledge (score 1–5, where 1 is the lowest)	1	16	11%
	2	33	23%
	3	24	16%
	4	37	25%
	5	36	25%

In this table, the demographics of the respondents are detailed (total *n* = 146) and expressed as a fraction and percentage

Respondents came from a wide range of ethnic backgrounds, but the majority were White/Caucasian (42/146, 29%) or Asian/Asian British (38/146, 26%) (Fig. S2). Participants report living in all four nations of the United Kingdom (across 48 regions), as well as the Isle of Man (ESM Supplementary Table S2). Those that reported residing in England (110/146, 75%) were frequently located in London, 16/146 (11%).

Most respondents reported education to a post-graduate (42/146, 35.6%) or undergraduate degree level (39/146, 26.7%) (Fig. S3).

### Computer and AI literacy

The majority of respondents described their computer skills as above average (84/146, 58%), including 35 respondents (35/146, 24%) reporting their computer skills as advanced. Half of the participants (73/146, 51%) rated their AI knowledge as average or less than average, whilst one quarter of respondents (36/146, 25%) described their AI knowledge as advanced.

Those with further education degrees were more likely to rate themselves as having above average knowledge about AI (53% (70/131)). In comparison, only 20% (3/15) of those without degrees rated themselves as having above average or advanced knowledge regarding AI. Male respondents were more likely to rate their AI knowledge as above average at 64% (46/72) compared to 36% (26/72) of female respondents.

### Opinions on AI for medical imaging

**Fig. 2:**
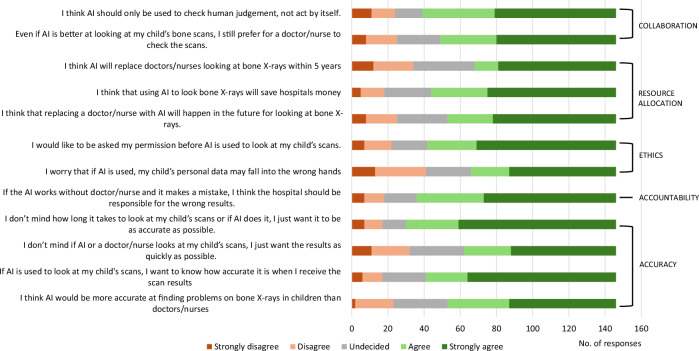
Bar chart demonstrating the opinions of respondents to the different questions relating to the use of AI for children’s imaging in this survey (with particular relevance to “bone X-rays”). Five key themes relating to the survey questions are shown on the right of the image

**Fig. 3:**
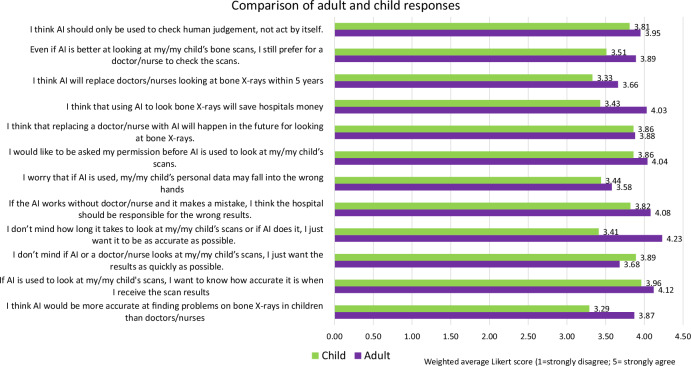
Bar chart showing the differences in average Likert score between adult and child respondents to the different questions relating to the use of AI for children’s imaging in this survey (with particular relevance to “bone X-rays”)

The full breakdown of all responses from the survey is outlined in Tables 2–5, Figs. 2 and 3, and Supplementary Table 3.

**Table 2:** FRACTURE survey—free text comments

Theme	Positive	Neutral	Negative
ACCURACY	…in the presence of classical fracture symptoms a well programmed AI can accurately identify fracture, I expect less fractures would be missed with AI being utilised in normal practice This would prevent major and minor fractures that could have been unseen and in total support This would ensure bone related issues are fully identified and fixed I think AI could potentially be used in conjunction with a live human professional improve the accuracy and speed of a correct diagnosis I think AI will be more accurate than clinicians for any type of scan and welcome their use in all diagnostic imaging		Expert live human judgement may be better at explaining novel phenomena than a computer programme.
ETHICAL ISSUES		It is imperative that a wide range of ethnicities, ages and genders are included in the studies. It is important that the data is as wide as possible.	Computers shouldn’t replace humans, as discussion in treatment is important The use of any tool should be evidence-based, and as far as I understand, the evidence hasn’t been collected yet.
RESOURCE ALLOCATION	“…can help the NHS to invest the saved cost elsewhere”		
COLLABORATION	I think that AI should be used especially in cases where doctors are struggling to spot a fracture I believe AI can do initial assessments, but these should always be checked by a clinician.	Validity of the results by a nurse or an AHP instead of a Radiologist can be explored. It could be used to double check I think the Dr/radiographer, etc., should make their diagnosis before seeing AI results, then review if there is a difference between the two.	I do not feel AI should replace human input.
GENERAL COMMENTS	AI is the change to medical technology Perhaps AI can help collect and keep track of the ‘odd’ cases (where no satisfactory healing outcome has been reached) for future study, so potentially new syndromes or conditions could be recognised in the future.	I don’t know enough about medicine to guess which particular cases will benefit from AI. I would like to see the evidence first before making my own Judgement.	

Free text responses from parents and carers regarding their opinions about how artificial intelligence (AI) should be used in hospitals for looking at children’s imaging. No specific comments relating to accountability were submitted

**Table 3:** Survey responses on use of AI for other pathologies (apart from fractures)

	Type	Likert scale (1 = strongly disagree; 5 = strongly agree)						Adult minus child’s average score difference
		1	2	3	4	5	Average score	
I think AI would be more accurate than doctors/nurses for finding								
Cancer on scans	Adult	10 (6.8%)	26 (17.8%)	31 (21.2%)	38 (26.0%)	41 (28.1%)	3.51	0.44^*^ *p* < 0.00001
	Child	4 (2.3%)	55 (32.2%)	52 (30.4%)	45 (26.3%)	15 (8.8%)	3.07	
Brain diseases on scans	Adult	6 (4.1%)	18 (12.3%)	40 (27.4%)	27 (18.5%)	55 (37.7%)	3.73	0.04 *p* = 0.08
	Child	7 (4.1%)	16 (9.4%)	47 (27.5%)	53 (31.0%)	47 (27.5%)	3.69	
Heart diseases on scans	Adult	3 (2.1%)	13 (8.95)	45 (30.8%)	26 (17.8%)	59 (40.4%)	3.86	0.37^*^ *p* = 0.002
	Child	7 (4.1%)	25 (14.6%)	53 (31.0%)	50 (29.2%)	36 (21.1%)	3.49	
Even if AI is better at looking for disease on my/my child’s scans, I’d still prefer for a doctor/nurse to check the scans								
For cancer	Adult	5 (3.4%)	8 (5.5%)	18 (12.3%)	35 (24.0%)	80 (54.8%)	4.21	0.29 *p* = 0.1
	Child	7 (4.1%)	23 (13.5%)	21 (12.3%)	46 (26.9%)	74 (43.3%)	3.92	
For brain diseases	Adult	5 (3.4%)	13 (8.9%)	21 (14.4%)	22 (15.1%)	85 (58.2%)	4.16	0.41^*^ *p* = 0.0001
	Child	6 (3.5%)	25 (14.6%)	31 (18.1%)	53 (31.0%)	56 (32.7%)	3.75	
For heart diseases	Adult	9 (6.2%)	14 (9.6%)	17 (11.6%)	24 (16.4%)	82 (56.2%)	4.07	0.36^*^ *p* = 0.0005
	Child	8 (4.7%)	25 (14.6%)	32 (18.7%)	50 (29.2%)	56 (32.7%)	3.71	
I would be more willing to have AI look at my/my child’s scans if they were checking for another disease (than bone problems)								
For cancer	Adult	6 (4.1%)	16 (11.0%)	36 (24.7%)	35 (24.0%)	53 (36.3%)	3.77	0.52^*^ *p* = 0.001
	Child	10 (5.8%)	35 (20.5%)	59 (34.5%)	36 (21.1%)	31 (18.1%)	3.25	
For brain diseases	Adult	8 (5.5%)	11 (7.5%)	42 (28.8%)	26 (17.8%)	59 (40.4%)	3.80	0.41^*^ *p* = 0.004
	Child	10 (5.8%)	27 (15.8%)	57 (33.3%)	40 (23.4%)	37 (21.6%)	3.39	
For heart diseases	Adult	9 (6.2%)	12 (8.2%)	39 (26.7%)	25 (17.1%)	61 (41.8%)	3.80	0.45^*^ *p* = 0.009
	Child	13 (7.6%)	28 (16.4%)	58 (33.9%)	31 (18.1%)	41 (24.0%)	3.35	

In this table, the number of responses from adults (*n* = 146) and percentages of responses to all AI-related questions with Likert scale answers are provided *n*, (%)

We compare our findings here with our previously published work on children’s views using the same survey questions adapted for children, previously published [7]. Where the Chi-squared test was significant (*p* < 0.05), an asterisk is shown in the final column

**Table 4:** Ethnicity subgroup analysis of survey responses to all AI-related questions

Question	Respondents who agree or strongly agree with this statement by ethnicity, fraction (%)					
	Asian	Black	Mixed	White	Prefer not to say	Total
ACCURACY						
I think AI would be more accurate at finding problems on bone X-rays in children than doctors/nurses	21/38 (55%)	16/23 (70%)	25/31 (81%)	25/42 (60%)	6/12 (50%)	93/146 (64%)
ACCURACY						
If AI is used to look at my/my child’s scans, I want to know how accurate it is when I receive the scan results	26/38 (68%)	15/23 (65%)	25/31 (81%)	34/42 (81%)	5/12 (42%)	105/146 (72%)
ACCURACY						
I don’t mind if AI or a doctor/nurse looks at my child’s scans, I just want the results as quickly as possible.	18/38 (47%)	17/23 (74%)	25/31 (81%)	22/42 (52%)	2/12 (17%)	84/146 (58%)
ACCURACY						
I don’t mind how long it takes to look at my child’s scans, or if AI does it, I just want it to be as accurate as possible.	27/38 (71%)	17/23 (74%)	26/31 (84%)	39/42 (93%)	7/12 (58%)	116/146 (79%)
ACCOUNTABILITY						
If the AI works without a doctor/nurse and it makes a mistake, I think the hospital should be responsible for the wrong results.	24/38 (63%)	15/23 (65%)	28/31 (90%)	36/42 (86%)	7/12 (58%)	110/146 (75%)
ETHICS						
I worry that if AI is used, my child’s personal data may fall into the wrong hands	21/38 (55%)	16/23 (70%)	24/31 (77%)	11/42 (26%)	8/12 (67%)	80/146 (55%)
ETHICS						
I would like to be asked for my permission before AI is used to look at my child’s scans.	28/38 (74%)	19/23 (83%)	26/31 (84%)	25/42 (60%)	6/12 (50%)	104/146 (71%)
RESOURCE ALLOCATION						
I think that replacing a doctor/nurse with AI will happen in the future for looking at bone X-rays.	23/38 (61%)	19/23 (83%)	23/31 (74%)	23/42 (55%)	5/12 (42%)	93/146 (64%)
RESOURCE ALLOCATION						
I think that using AI to look at bone X-rays will save hospitals money	28/38 (74%)	16/23 (70%)	26/31 (84%)	27/42 (64%)	5/12 (42%)	102/146 (70%)
RESOURCE ALLOCATION						
I think AI will replace doctors/nurses looking at bone X-rays within 5 years	19/38 (50%)	17/23 (74%)	24/31 (77%)	12/42 (29%)	6/12 (50%)	78/146 (53%)
COLLABORATION						
Even if AI is better at looking at/my child’s bone scans, I still prefer for a doctor/nurse to check the scans.	24/38 (63%)	16/23 (70%)	25/31 (81%)	26/42 (62%)	6/12 (50%)	97/146 (66%)
COLLABORATION						
I think AI should only be used to check human judgment, not act on itself.	24/38 (63%)	20/23 (87%)	26/31 (84%)	31/42 (74%)	6/12 (50%)	107/146 (73%)

In this table, the number of respondents who agree or strongly agree with AI-related statements from each ethnic group (total *n* = 146) expressed as a fraction and percentage are provided

**Table 5:** Age subgroup analysis of survey responses to all AI-related questions

Question	Respondents who agree or strongly agree with this statement by age, fraction (%)			
	21–40 years of age	41–50 years of age	51 and above years of age	Total
ACCURACY				
I think AI would be more accurate at finding problems on bone X-rays in children than doctors/nurses	32/53 (60%)	33/49 (67%)	28/44 (64%)	93/146 (64%)
ACCURACY				
If AI is used to look at my/my child’s scans, I want to know how accurate it is when I receive the scan results	35/53 (66%)	37/49 (76%)	33/44 (75%)	105/146 (72%)
ACCURACY				
I don’t mind if AI or a doctor/nurse looks at my child’s scans, I just want the results as quickly as possible.	25/53 (47%)	31/49 (63%)	28/44 (64%)	84/146 (58%)
ACCURACY				
I don’t mind how long it takes to look at my/my child’s scans, or if AI does it, I just want it to be as accurate as possible.	38/53 (72%)	41/49 (84%)	37/44 (84%)	116/146 (79%)
ACCOUNTABILITY				
If the AI works without a doctor/nurse and it makes a mistake, I think the hospital should be responsible for the wrong results.	37/53 (70%)	38/49 (78%)	35/44 (80%)	110/146 (75%)
ETHICS				
I worry that if AI is used, my child’s personal data may fall into the wrong hands	26/53 (49%)	25/49 (51%)	29/44 (66%)	80/146 (55%)
ETHICS				
I would like to be asked for my permission before AI is used to look at my child’s scans.	33/53 (62%)	38/49 (78%)	33/44 (75%)	104/146 (71%)
RESOURCE ALLOCATION				
I think that replacing a doctor/nurse with AI will happen in the future for looking at bone X-rays.	27/53 (51%)	34/49 (69%)	32/44 (73%)	93/146 (64%)
RESOURCE ALLOCATION				
I think that using AI to look at bone X-rays will save hospitals money	35/53 (66%)	33/49 (67%)	34/44 (77%)	102/146 (70%)
RESOURCE ALLOCATION				
I think AI will replace doctors/nurses looking at bone X-rays within 5 years	24/53 (45%)	28/49 (57%)	26/44 (59%)	78/146 (53%)
COLLABORATION				
Even if AI is better at looking at my/my child’s bone scans, I still prefer for a doctor/nurse to check the scans.	29/53 (55%)	39/49 (80%)	29/44 (66%)	97/146 (66%)
COLLABORATION				
I think AI should only be used to check human judgment, not act on its.	35/53 (66%)	37/49 (76%)	35/44 (80%)	107/146 (73%)

In this table, the number of respondents who agree or strongly agree with AI-related statements from each ethnic group (total *n* = 146) expressed as a fraction and percentage are provided

### Accuracy

**Fig. 4:**
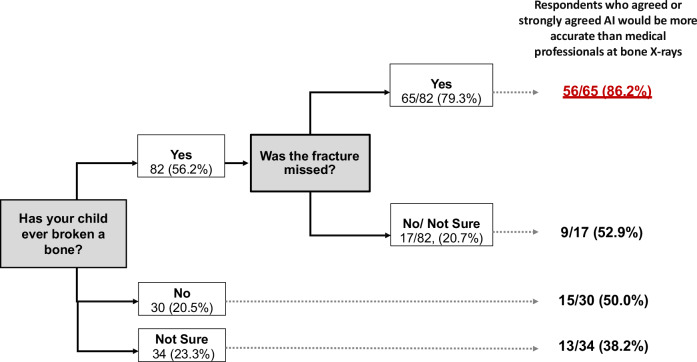
Flow chart demonstrating the differences in the proportion of respondents’ opinions regarding AI accuracy for bone X-rays relative to their own personal experience of sustaining a fracture

More than half of the respondents (56%, 82/146) reported that their child (or a child they frequently care for) had previously broken a bone (Fig. 4). Of these, 79% (65/82) reported that the fracture was initially missed on X-ray.

When asked about the accuracy of AI for detecting bone fractures, 64% (93/146) agreed or strongly agreed that AI would be more accurate than doctors/nurses. However, in a subset of respondents who reported the fracture had been missed on the initial X-ray, 86% (56/65) agreed or strongly agreed that AI would be more accurate.

Male (55/72, 76%) respondents were more likely to agree or strongly agree that AI was more accurate than doctors/nurses when interpreting bone imaging, compared to 50% (36/72) of female respondents. Nevertheless, half (84/146, 58%) of respondents agreed or strongly agreed that they do not mind whether AI or a healthcare professional interprets a scan, they just want a result quickly. A vast majority (116/146, 79%) agreed or strongly agreed that they did not care how long it took to get a scan result as long as it was as accurate as possible, regardless of who assessed the examination. Adults were more likely to prioritise accuracy over speed or human interpretation than in children and young people (average Likert score 4.23 in adults vs 3.41 in CYAP; *p* < 0.05) [7].

If the child had previously suffered a fracture, the parents and carers were more likely to agree or strongly agree that AI would be more accurate than doctors/nurses at looking at bone radiographs than respondents without a fracture history (65/82, 79% compared to 28/64, 44%, *p* < 0.05). Similarly, they don’t mind whether the AI or healthcare professional interprets the scan, they just want the results to be as accurate as possible (77/82, 94% agreed or strongly agreed compared to 39/64, 61% without fracture history, *p* < 0.05), and to get the results as quickly as possible (59/82, 72% compared to 25/64, 39%, *p* < 0.05).

A similar trend was seen in respondents with higher-than-average self-reported AI knowledge. They were more likely to agree or strongly agree that AI would be more accurate than healthcare professionals compared to those with average or below average self-reported AI knowledge (58/73, 79% vs 35/73, 48%, *p* < 0.05, respectively). They also prioritised accuracy and speed over who looked at the scans (66/73, 90% vs 50/73, 68%, *p* < 0.05 and 51/73, 70% vs 33/73, 45%, *p* < 0.05, respectively).

Respondents who were educated to below an undergraduate level were more likely to agree/strongly agree that they don’t mind who looks at the scans, but just want the results as quickly as possible, compared to those with at least an undergraduate degree (26/35, 74% compared to 58/111, 52%, respectively, *p* < 0.05).

Most respondents agreed or strongly agreed (105/146, 72%) with the statement that they would like to know how accurate the AI used is when they receive scan results. Parents and carers were more likely to want to know how accurate the AI is when receiving scan results than children (average Likert score 4.12 in adults vs 3.96 in CYAP, *p* = 0.01) [7].

### Accountability

Overall, most respondents agreed or strongly agreed that if AI were used without human supervision, the hospital should be held responsible for any inaccurate results produced (110/146, 75% agreed or strongly agreed with this). This is in comparison with 66% (113/171) of CYAP respondents in our prior study [7]. No specific free-text comments relating to accountability were submitted.

### Ethical issues

Male respondents (52/72, 72%) were twice as likely as female respondents (26/72, 36%) to agree or strongly agree that they were concerned about their child’s data falling into the wrong hands if AI is used. In general, over half of respondents (81/149, 55%) report being slightly worried about the safety of their child’s data when AI is used, and this was less of a concern in CYAP (average Likert score 3.56 in adults vs 3.44 in CYAP; *p* < 0.05) [7].

Most respondents agree or strongly agree (104/146, 71%) that they would like to be asked permission before AI is used for interpretation of their child’s scans, which was less strongly voiced in CYAP but not statistically significant (average Likert score of 4.04 in adults vs 3.86 in children, *p* = 0.06) [7].

### Resource allocation

Most parent/carer respondents agreed or strongly agreed (102/146, 70%, score 4.03) with the statement that using AI to look at bone scans would save hospitals money, and many respondents (93/146, 64%) agree or strongly agree that AI will replace doctors/nurses in the future for looking at bone X-rays, while just over half (78/146, 53%) believe this will happen within 5 years. Black (19/23, 83%) and mixed (24/31, 74%) ethnicities are more likely to agree or strongly agree that AI will replace doctors/nurses for looking at bone X-rays at some point in the future.

### Human/machine collaboration

A large proportion of respondents (107/146, 73%) agree or strongly agree that AI should only be used to check human judgment, not act on its own. Male participants (59/72, 82%) more frequently agreed or strongly agreed about the importance of AI working in conjunction with human judgment than female participants (47/72, 65%).

The majority of participants agreed or strongly agreed that even if AI was better at interpreting imaging, they would still prefer a doctor/nurse to check the scans (97/146, 66%). This was similar amongst both male (51/72, 70%) and female (45/72, 63%) participants. Compared to CYAP, parents/carers felt more strongly about this sentiment (average Likert score 3.89 vs 3.51 in CYAP; *p* < 0.05) [7].

### Use of AI in imaging

Slightly over half of participants agreed or strongly agreed with the statement that AI would be more accurate than doctors/nurses for finding cancer (79/146, 54%), brain (82/146, 56%) or heart (85/146, 58%) disease on their child’s scans. Comparatively, fewer CYAP expressed this sentiment for finding cancer (60/171, 35%) and heart disease (86/171, 50%) [7].

**Fig. 5:**
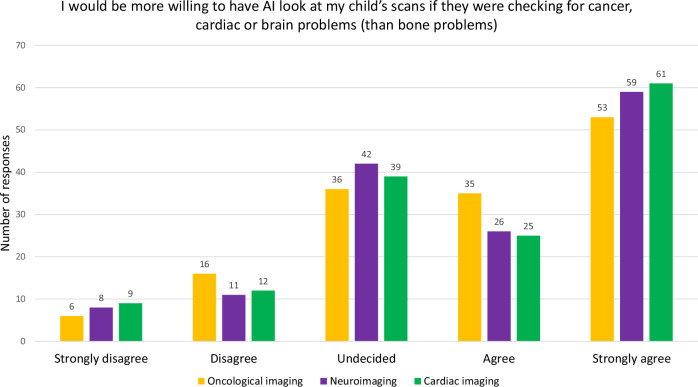
Bar chart showing the differences in opinions amongst the respondents about their willingness for AI to be used to interpret imaging for other diseases such as cancer, cardiac or brain diseases. Respondents felt positively about AI, looking at their child’s cancer, cardiac or brain scans in comparison to bone

**Fig. 6:**
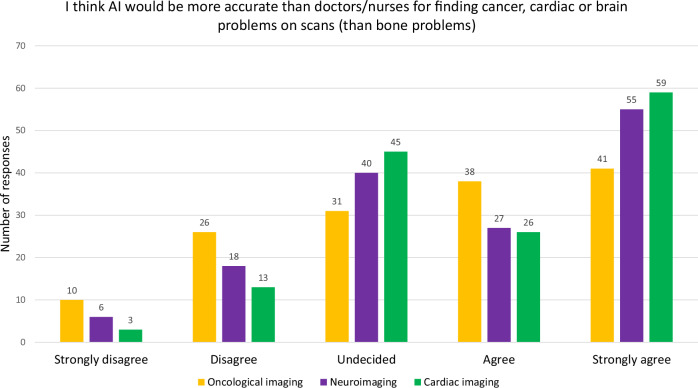
Bar chart showing the differences in opinions amongst the respondents about the perception of AI accuracy for detecting other diseases, such as cancer, cardiac or brain diseases. Most respondents felt that AI was less likely to be accurate at identifying cancer than brain or heart diseases than healthcare professionals

Respondents agreed or strongly agreed that they preferred human supervision of the AI when looking at cancer (115/146, 79% agree or strongly agree), brain (107/146, 73% agree or strongly agree) and heart (106/146, 73% agree or strongly agree) scans (average range of Likert scores: 4.07–4.21), compared to bone scans (score 3.51). Parent/carer views appeared to be more strongly expressed than those of CYAP (score 3.71–3.92 for cancer/brain/heart scans) [7]. See Table 3 and Figs. 5 and 6.

## Discussion

This study revealed that parents and carers felt that AI would be more accurate and efficient than healthcare professionals when looking at paediatric imaging. Despite this, respondents expressed a strong preference for AI integration with supervision of healthcare professionals. Respondents also expressed a strong desire to know when AI is used for their child’s imaging, to be informed how accurate the technology is before its use, and to be asked for permission before AI is used to interpret their child’s scans.

Our survey also found differences in the perception of AI and its use in healthcare between genders, ethnicities, age groups and educational attainment levels e.g. respondents from Black and Mixed ethnic backgrounds were more likely to believe that AI would be more accurate than doctors when looking at bone X-rays, but also the most likely to prefer human supervision of AI; and men were more likely to believe data would be mishandled than women. These findings illustrate the importance of involving a variety of stakeholders in surveys to accurately represent the varied opinions of parents/carers.

While this survey was targeted at parents and carers, our previously published comparator study using the same questions (adapted for a younger audience) investigated the opinions of CYAP regarding AI use for their imaging [7]. The main statistically significant differences between the two groups were that parents/carers were more likely to agree that AI would be more accurate than doctors/nurses, more likely to expect information about the AI being used (e.g. accuracy), worry about data being misused, and to advocate for human-AI collaboration. These results demonstrate CYAP showing more cautious responses than adults to accuracy and ethical questions around AI, which could be attributed to their more likely having grown up with technology and having more awareness of its limitations. Both groups, however, placed equally high value on being asked for consent prior to the use of AI. Both groups felt it was likely that AI would replace doctors/nurses for the interpretation of bone X-rays in the future.

There are few publications in the existing literature that explore the perceptions of parents and carers for AI use in paediatric imaging, and none that directly compare attitudes from CYAP with parents/carers. Nevertheless, in the literature, we found many similar themes in studies evaluating views of parents/carers that echo our results. An observational study by Roberts et al [6] used a cross-sectional survey to explore the attitudes of 184 parents/carers towards AI for management of orthopaedic medical images in paediatric patients. Similar to our study, they found that respondents preferred AI to assist in the diagnosis of fractures but not to work independently. In another study investigating views of AI in healthcare in the United States, over 70% of 804 parents of paediatric patients surveyed were open to AI use in healthcare, but only if there was evidence of AI accuracy, and AI was not used to replace healthcare professionals [13].

Another study by Ramgopal et al [14] received 1620 responses to a citywide household survey in the United States to explore parental perception on the use of AI in paediatric acute care. Yet again, their survey demonstrated that parents wanted to be notified when AI was used in their child’s care, with almost 30% of respondents saying this was ‘extremely important’. Haley et al [5] also used a cross-sectional survey to investigate the opinions of parents/carers of children in a Texan hospital, and they found that men were more open to AI involvement in various aspects of their child’s care, from diagnosis of cancer to administering medications. Our survey revealed that men were also more likely to believe that AI would be more accurate than healthcare professionals than women, but they were also more wary about data privacy. This distinction is important as it may mean that when consulting male carers/parents, a greater focus may need to be placed on assuring them of data concerns, whereas female carers/parents may appreciate a more detailed discussion regarding the performance of the AI tool.

Despite the many strengths of our study, our survey had some limitations. Firstly, the open recruitment strategy for an online survey likely introduced bias in the types of respondents as they required access to the internet, the ability to use a digital device and a good understanding of the English language. This is reflected in the fact that most respondents have a higher education qualification, with 36% having a postgraduate degree. Whilst this introduced some bias, there was considerable ethnic, gender and geographic diversity amongst the respondents. Additionally, there were only 146 responses, which may not be reflective of the entire population.

Interestingly, the proportion of ‘missed fractures’ reported by parents/carers in our survey was higher than expected for a general population (79% in this survey, in comparison to an estimated 5–19% missed paediatric fractures by emergency clinicians) [15–18]. This high ‘miss rate’ could be attributed to the fact that we recruited parents/carers via many channels, including the ‘Brittle Bone Charity’, which supports patients with osteogenesis imperfecta.

Finally, this survey concentrated on opinions about AI use primarily in the diagnosis of musculoskeletal diseases in paediatric imaging. Whilst we did ask questions regarding other diseases (e.g. heart, brain diseases and cancer), we did not ask respondents their own experiences with these conditions, like we did for fracture detection. The reason for this was to gain a general overview of whether different contexts matter to parents/carers. Further research could delve deeper into these disease categories.

In the future, research in this field could involve focus groups or individual interviews with parents and carers to allow closer inspection of some of the worries regarding ethical issues, such as how to best provide information about AI use for imaging to patients. This will be especially important given that many respondents dislike the idea of autonomous AI, but do admit that they see this as a future likelihood in healthcare.

Overall, CYAP and their parents/carers appear to have broadly similar opinions regarding perceptions of AI use in paediatric imaging, with parents/carers having slightly stronger views about the improved accuracy of AI, accountability, need for information about the AI being used, worry about data being misused, and approval for human-AI collaboration. To instil trust in AI tools, radiology departments should provide literature regarding the AI they intend to introduce, promote transparency for performance and details regarding accountability and safety measures, and explore a way to consent patients for use where possible. Future research could be conducted with focus groups to explore more comprehensively the variety of perspectives uncovered by our survey, especially around best practices for healthcare professionals and patient/parent stakeholders to engage with each other.

## Supplementary information

ELECTRONIC SUPPLEMENTARY MATERIAL

## Abbreviations

AI: Artificial intelligence
GOSH YPAG: Great Ormond Street Hospital Young Persons’ Advisory Group for research
PPIE: Patient and public involvement and engagement
YPAG: Young Persons Advisory Group for research
