# Supplementary material for: Parental and carer views on the use of AI in imaging for children: a national survey
Source: Insights Imaging. 2025 Aug 9;16:172. doi: 10.1186/s13244-025-02021-6 (PMC12335414; doi:10.1186/s13244-025-02021-6)

Parental and Carer Views on the Use of AI in Imaging for Children: A National Survey

ELECTRONIC SUPPLEMENTARY MATERIAL

Contents

Title:	Page:
Table S1: Survey Questionnaire	2
Table S2: Location of survey respondents (in alphabetic order)	5
Table S3: **FRACTURE Survey Summarised Total Responses**	6
Figure S1: Bar chart demonstrating the spread of ages of respondents to our survey	8
Figure S2: Pie chart depicting the self-reported ethnicity of the respondents (n, %) in this survey	8
Figure S3: Bar chart depicting the self-reported highest level of educational attainment of the respondents in this survey	9

Supplementary Table S1.
FRACTURE Survey: Should AI be used to make decisions in hospitals?

QUESTION	RESPONSE ALLOWED
What is your gender?	Female; Male; Non-binary; Prefer not to say; Other
What is your age? (years old)	20 or below; 21-30; 31-40; 41-50; 51-60; 61-70; Prefer not to say; Other
What is your ethnicity?	White/Caucasian<br>Mixed/Multiple ethnic groups<br>Asia/ Asian British<br>Black/ African/ Caribbean/ Black British<br>Prefer not to say<br>Other
Where do you currently live (city, country)?	Open-Ended Response
What best describes your current/ previous caring situation with children? Please tick as many that apply to you. Each option includes adoptive or 'step' carers too.	Parent/ Grandparent/ Guardian/ Foster Carer/ Aunt/ Uncle/ Grandaunt/ Granduncle/ Work with children in my job role/ Other
What is the highest degree or level of education you have completed?	Never attended formal education; Primary/elementary school; High school/ secondary school; Associate/ trade/ vocational degrees; Undergraduate university degree (e.g. BA, BSc); Postgraduate university degree (e.g. MSc, MA); Professional degrees (e.g. medical, engineering)
How do you rate your computer skills?	Likert Scale 1 – 5; 1 = not very good, 5 = excellent
How much knowledge do you already have about artificial intelligence in general?	Likert Scale 1 – 5; 1 = nothing at all, 5 = a lot
Has your child ever broken a bone before?	Yes; No; Not sure
Was the broken bone initially missed on Xray tests?	Yes; No; Not sure; Not applicable (child not broken a bone)
I think AI would be more accurate at finding problems on bone X-rays in children than doctors/nurses.	Likert Scale 1 – 5; 1 = strongly disagree, 5 = strongly agree

Even if AI is better at looking at my child’s bone scans, I still prefer for a doctor/nurse to check the scans.	Likert Scale 1 – 5; 1 = strongly disagree, 5 = strongly agree
I think AI should only be used to check human judgement, not act by itself.	Likert Scale 1 – 5; 1 = strongly disagree, 5 = strongly agree
I worry that if AI is used, my child’s personal data may fall into the wrong hands	Likert Scale 1 – 5; 1 = strongly disagree, 5 = strongly agree
If the AI works without doctor/nurse and it makes a mistake, I think the hospital should be responsible for the wrong results.	Likert Scale 1 – 5; 1 = strongly disagree, 5 = strongly agree
I would like to be asked my permission before AI is used to look at my child’s scans.	Likert Scale 1 – 5; 1 = strongly disagree, 5 = strongly agree
If AI is used to look at my child's scans, I want to know how accurate it is when I receive the scan results	Likert Scale 1 – 5; 1 = strongly disagree, 5 = strongly agree
I don’t mind if AI or a doctor/nurse looks at the child’s scans, I just want the results as quickly as possible.	Likert Scale 1 – 5; 1 = strongly disagree, 5 = strongly agree
I don’t mind how long it takes to look at my child’s scans or if AI does it, I just want it to be as accurate as possible.	Likert Scale 1 – 5; 1 = strongly disagree, 5 = strongly agree
I think that replacing a doctor/nurse with AI will happen in the future for looking at bone X-rays.	Likert Scale 1 – 5; 1 = strongly disagree, 5 = strongly agree
I think that using AI to look bone X-rays will save hospitals money	Likert Scale 1 – 5; 1 = strongly disagree, 5 = strongly agree
Are there any extra comments or opinions you wish to make? For example, were there certain views that you feel particularly strongly about that you wish to explain in detail here?	Open-Ended Response
1. I think AI would be more accurate than doctors/nurses for finding cancer on scans	Likert Scale 1 – 5; 1 = strongly disagree, 5 = strongly agree

2. Even if AI is better at looking for cancer on my child’s scans, I'd still prefer for a doctor/nurse to check the scans.	Likert Scale 1 – 5; 1 = strongly disagree, 5 = strongly agree
3. I would be more willing to have AI look at my child's scans if they were checking for cancer (than bone problems)	Likert Scale 1 – 5; 1 = strongly disagree, 5 = strongly agree
1. I think AI would be more accurate than doctors/nurses for finding brain diseases on scans	Likert Scale 1 – 5; 1 = strongly disagree, 5 = strongly agree
2. Even if AI is better at looking for brain diseases on my child’s scans, I'd still prefer for a doctor/nurse to check the scans.	Likert Scale 1 – 5; 1 = strongly disagree, 5 = strongly agree
3. I would be more willing to have AI look at my child’s scans if they were checking for brain diseases (than bone problems)	Likert Scale 1 – 5; 1 = strongly disagree, 5 = strongly agree
1. I think AI would be more accurate than doctors/nurses for finding heart diseases on scans	Likert Scale 1 – 5; 1 = strongly disagree, 5 = strongly agree
2. Even if AI is better at looking for heart diseases on my child’s scans, I'd still prefer for a doctor/nurse to check the scans.	Likert Scale 1 – 5; 1 = strongly disagree, 5 = strongly agree
3. I would be more willing to have AI look at my child’s scans if they were checking for heart diseases (than bone problems)	Likert Scale 1 – 5; 1 = strongly disagree, 5 = strongly agree
Are there any comments or opinions you wish to make? For example, were there certain views that you feel particularly strongly about that you wish to explain in detail here?	Open-Ended Response

Supplementary Table 2:
Location of survey respondents (in alphabetic order)

County/Region:	n (%)	County/Region:	n (%)
Region not specified	1 (0.68%)	Isle of Man	1 (0.68%)
**England**			
England, region not specified	1 (0.68%)	Herefordshire	1 (0.68%)
Avon	8 (5.48%)	Kent	2 (1.37%)
Berkshire	1 (0.68%)	Lancashire	6 (4.11%)
Buckinghamshire	1 (0.68%)	Lincolnshire	1 (0.68%)
Cambridgeshire	9 (6.16%)	London	16 (11.0%)
Cheshire	3 (2.05%)	Merseyside	3 (2.05%)
Cornwall	1 (0.68%)	Norfolk	3 (2.05%)
Cumbria	1 (0.68%)	North Yorkshire	5 (3.42%)
Derbyshire	3 (2.05%)	Nottinghamshire	1 (0.68%)
Devon	3 (2.05%)	Oxfordshire	7 (4.79%)
Durham	4 (2.74%)	Shropshire	1 (0.68%)
East Sussex	3 (2.05%)	South Yorkshire	4 (2.74%)
Essex	4 (2.74%)	Surrey	2 (1.37%)
Hampshire	2 (1.37%)	West Midlands	3 (2.05%)
Greater Manchester	1 (0.68%)	West Sussex	3 (2.05%)
Hereford and Worcester	2 (1.37%)	West Yorkshire	5 (3.42%)
**Wales**			
Wales, region not specified	1 (0.68%)	Pembrokeshire	1 (0.68%)
Clwyd	1 (0.68%)	South Glamorgan	3 (2.05%)
Gwent	3 (2.05%)	West Glamorgan	2 (1.37%)
Gwynedd	1 (0.68%)		
**Scotland**			
Scotland, region not specified	1 (0.68%)	Fife	1 (0.68%)

Aberdeenshire	3 (2.05%)	Glasgow	3 (2.05%)
Angus	4 (2.74%)	Perthshire	5 (3.42%)
Edinburgh	1 (0.68%)		
**Northern Ireland**			
City of Belfast	1 (0.68%)	County Down	2 (1.37%)
County Armagh	1 (0.68%)		

Supplementary Table 3. FRACTURE Survey Summarised Total Responses
In this table, the number of responses from adults (n = 146) and percentages of responses to all AI related questions with Likert scale answers are provided n, (%). The final column presents the weighted average score amongst all responses.

We compare our findings here with our previously published work on children's views (n = 171) using the same survey questions adapted for children, previously published [7]. Where the Chi squared test was significant (p<0.05) an asterisk is shown in the final column.

Question	Likert Scale (1 = strongly disagree; 5 = strongly agree)							Adult minus child's avg score difference (p value)
	Type	1	2	3	4	5	Avg score	
**ACCURACY** I think AI would be more accurate at finding problems on bone X-rays in children than doctors/nurses	Adult	2 (1.4%)	21 (14.4%)	30 (20.5%)	34 (23.3%)	59 (40.4%)	3.87	**0.58* p<0.00001**
	Child	2 (1.2%)	39 (22.8%)	57 (33.3%)	54 (31.6%)	19 (11.1%)	3.29	
**ACCURACY** If AI is used to look at my/my child's scans, I want to know how accurate it is when I receive the scan results	Adult	6 (4.1%)	11 (7.5%)	24 (16.4%)	23 (15.8%)	82 (56.2%)	4.12	**0.16* p = 0.01**
	Child	4 (2.3%)	18 (10.5%)	27 (15.8%)	53 (31.0%)	69 (40.4%)	3.96	
**ACCURACY** I don't mind if AI or a doctor/nurse looks at my/my child's scans, I just want the results as quickly as possible.	Adult	11 (7.5%)	21 (14.4%)	30 (20.5%)	26 (17.8%)	58 (39.7%)	3.68	**-0.21* p = 0.002**
	Child	4 (2.3%)	39 (22.8%)	52 (30.4%)	35 (20.5%)	41 (24.0%)	3.89	
**ACCURACY** I don't mind how long it takes to look at my/my child's scans or if AI does it, I just want it to be as accurate as possible.	Adult	7 (4.8%)	10 (6.8%)	13 (8.9%)	29 (19.9%)	87 (59.6%)	4.23	**0.82* p = 0.0002**
	Child	2 (1.2%)	22 (12.9%)	34 (19.9%)	47 (27.5%)	66 (38.6%)	3.41	
**ACCOUNTABILITY** If the AI works without doctor/nurse and it makes a mistake, I think the hospital	Adult	7 (4.8%)	11 (7.5%)	18 (12.3%)	37 (25.3%)	73 (50.0%)	4.08	**0.26* p = 0.002**
	Child	2 (1.2%)	23 (13.5%)	33 (19.3%)	58 (33.9%)	55 (32.2%)	3.82	

should be responsible for the wrong results.								
**ETHICS** I worry that if AI is used, my/my child's personal data may fall into the wrong hands	Adult	13 (8.9%)	28 (19.2%)	25 (17.1%)	21 (14.4%)	59 (40.4%)	3.58	**0.14* p = 0.00004**
	Child	4 (2.3%)	42 (24.6%)	37 (21.6%)	51 (29.8%)	37 (21.6%)	3.44	
**ETHICS** I would like to be asked my permission before AI is used to look at my/my child's scans.	Adult	7 (4.8%)	15 (10.3%)	20 (13.7%)	27 (18.5%)	77 (52.7%)	4.04	0.18 p = 0.06
	Child	4 (2.3%)	21 (12.3%)	37 (21.6%)	42 (24.6%)	67 (39.2%)	3.86	
**RESOURCE ALLOCATION** I think that replacing a doctor/nurse with AI will happen in the future for looking at bone X-rays.	Adult	8 (5.5%)	17 (11.6%)	28 (19.2%)	25 (17.1%)	68 (46.6%)	3.88	0.02 p = 0.25
	Child	4 (2.3%)	21 (12.3%)	37 (21.6%)	42 (24.6%)	67 (39.2%)	3.86	
**RESOURCE ALLOCATION** I think that using AI to look bone X-rays will save hospitals money	Adult	5 (3.4%)	13 (8.9%)	26 (17.8%)	31 (21.2%)	71 (48.6%)	4.03	**0.60* p = 0.0001**
	Child	9 (5.3%)	34 (19.9%)	43 (25.1%)	44 (25.7%)	41 (24.0%)	3.43	
**RESOURCE ALLOCATION** I think AI will replace doctors/nurses looking at bone X-rays within 5 years	Adult	12 (8.2%)	22 (15.1%)	34 (23.3%)	13 (8.9%)	65 (44.5%)	3.66	**0.33* p = 0.01**
	Child	14 (8.2%)	39 (22.8%)	41 (24.0%)	30 (17.5%)	47 (27.5%)	3.33	
**COLLABORATION** Even if AI is better at looking at my/my child's bone scans, I still prefer for a doctor/nurse to check the scans.	Adult	8 (5.5%)	17 (11.6%)	24 (16.4%)	31 (21.2%)	66 (45.2%)	3.89	**0.38* p = 0.00007**
	Child	2 (1.2%)	42 (24.6%)	35 (20.5%)	50 (29.2%)	42 (24.6%)	3.51	
**COLLABORATION** I think AI should only be used to check human judgement, not act by itself.	Adult	11 (7.5%)	13 (8.9%)	15 (10.3%)	40 (27.4%)	67 (45.9%)	3.95	**0.14* p = 0.008**
	Child	4 (2.3%)	19 (11.1%)	38 (22.2%)	49 (28.7%)	61 (35.7%)	3.81	

Figure S1
Bar chart demonstrating the spread of ages of respondents to our survey (n=146)

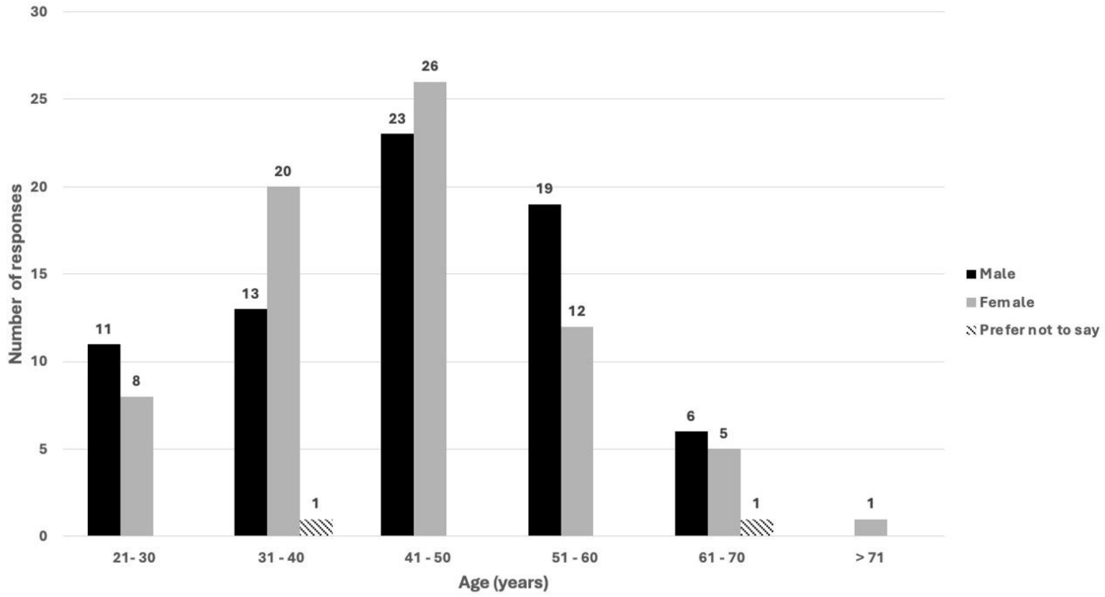

Figure S2
Pie chart depicting the self-reported ethnicity of the respondents (n, %) in this survey (n = 146)

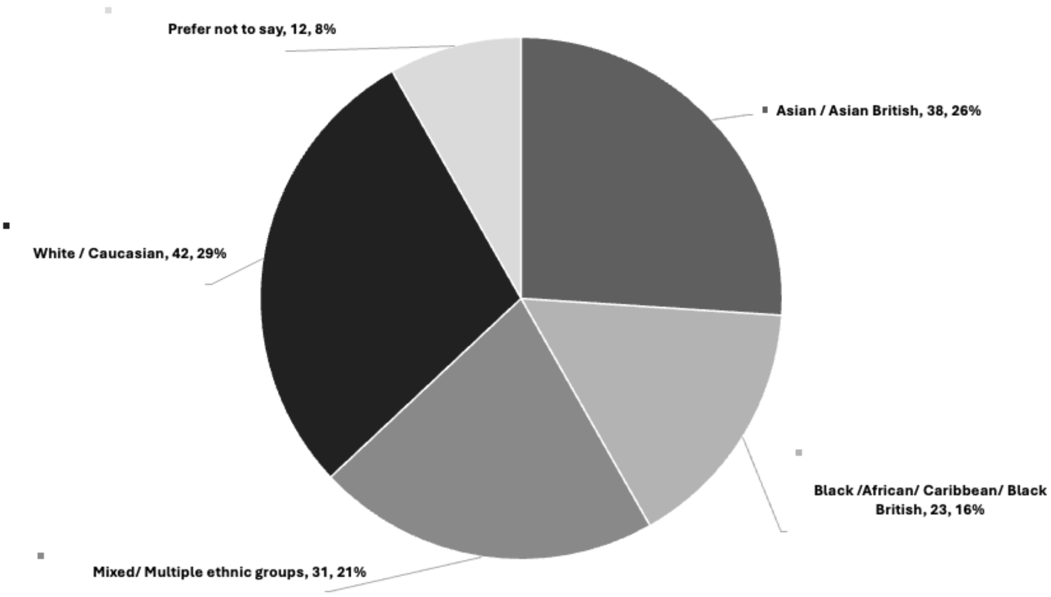

Figure S3
Bar chart depicting the self-reported highest level of educational attainment of the respondents in this survey (n = 146)

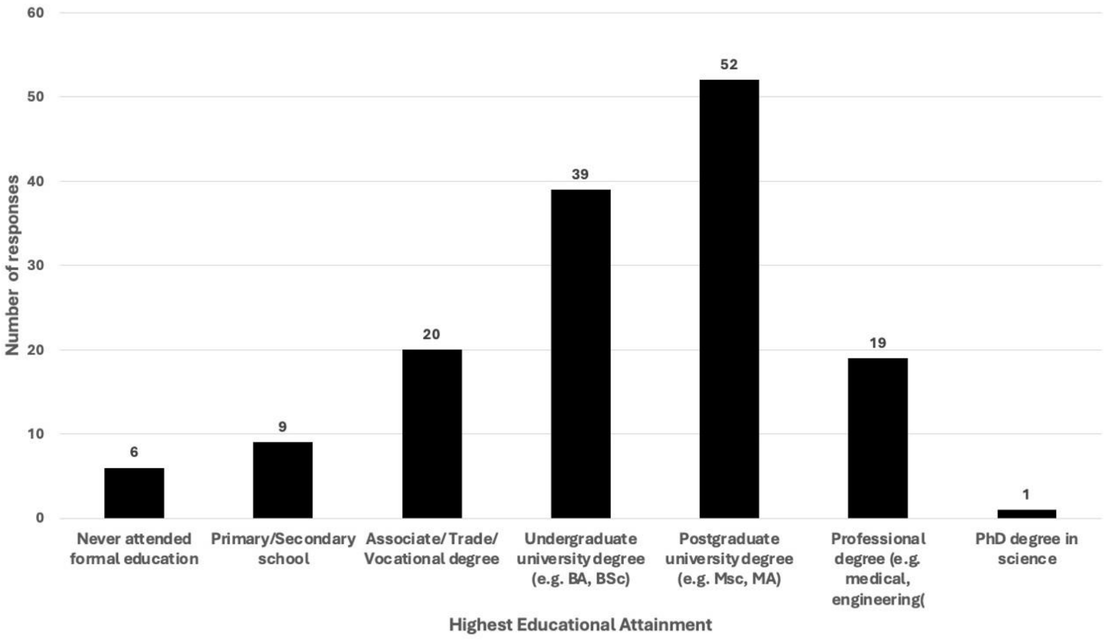

Supplement: Supplementary file 1 — ELECTRONIC SUPPLEMENTARY MATERIAL [file 13244_2025_2021_MOESM1_ESM.pdf]
